# Wo steht mir der Kopf? – Herausforderungen von berufsbegleitend Studierenden während der COVID-19-Pandemie

**DOI:** 10.1007/s16024-021-00351-1

**Published:** 2021-04-20

**Authors:** Andrea Limarutti, Simone Sigrid Flaschberger, Eva Mir

**Affiliations:** 1 Studienbereich Gesundheit und Soziales, Fachhochschule Kärnten gemeinnützige Privatstiftung, Hauptplatz 12, 9560 Feldkirchen i.K., Österreich; 2 Institut für Humanwissenschaften, Pädagogische Hochschule Schwäbisch Gmünd, Oberbettringer Str. 200, Schwäbisch Gmünd, 73525 Deutschland

**Keywords:** Gesundheitsberufe, Qualitative Inhaltsanalyse, Vereinbarkeit Studium und Beruf, Kohärenzgefühl, Studierendengesundheit, Health professionals, Qualitative content analysis, Compatibility of studies and work, Sense of coherence, Students’ health

## Abstract

**Hintergrund:**

Aufgrund der COVID-19-Pandemie waren Hochschulen gezwungen, schnellstmöglich auf Onlinelehre umzustellen. Berufsbegleitend Studierenden, welche etwa im Gesundheitsbereich tätig sind, muss die Vereinbarkeit von der 100 %igen Umstellung auf E‑Learning und vollen Anwesenheit in einem systemerhaltenden Beruf gelingen.

**Zielsetzung:**

Ziel der Studie ist es, die Herausforderungen von im Gesundheitsbereich tätigen berufsbegleitend Studierenden zu explorieren, Veränderungsnotwendigkeiten aufzuzeigen und Handlungsempfehlungen für die Hochschule abzuleiten.

**Methode:**

Mittels Onlinefragebogen und offenem Antwortformat wurden Herausforderungen, Vor- und Nachteile, welche sich durch das E‑Learning ergeben, und Verbesserungsvorschläge an der Fachhochschule Kärnten (FHK) erhoben. Die Daten wurden mittels qualitativer Inhaltsanalyse nach Mayring ausgewertet.

**Ergebnisse:**

Die wohl größten Herausforderungen, mit denen berufsbegleitend Studierende zu kämpfen haben, sind das selbstorganisierte Lernen bzw. die (termingerechte) Bewältigung der, oft als zu umfangreich empfundenen, Arbeitsaufträge und das selbstständige Erarbeiten von Lehrinhalten. Weiters berichten die Studierenden über Schwierigkeiten in der Vereinbarkeit von E‑Learning mit Beruf und/oder Familie.

**Schlussfolgerung:**

Die empfundenen Herausforderungen sind grundsätzlich nicht neu, aber unter COVID-19 bedeutsamer denn je, da noch zusätzliche familiäre Verpflichtungen, aber auch Unsicherheiten im Arbeitsalltag dazukommen. Gerade jetzt sollten Lernumwelten, wie sie Hochschulen berufsbegleitend Studierenden derzeit online bieten, als „caring spaces“ verstanden werden. Neben neuen didaktischen Konzepten sollte auch in die Förderung von Selbst- und Sozialkompetenzen der Studierenden investiert werden.

## Abstract (en)

**Background:**

Due to the COVID-19 pandemic, universities had to switch to online teaching as quickly as possible. Part-time students who work in the healthcare sector have to manage the balancing act between an invariably online education and a mandatory physical attendance in a profession that maintains the system.

**Aim:**

The aim of the study was to explore the challenges faced by part-time students working in the healthcare sector, to identify the need for change and to derive recommendations for action for the university.

**Methods:**

The data were collected using an open response online questionnaire. The obtained data were evaluated according to the qualitative content analysis of Mayring.

**Results:**

Probably the greatest challenges that part-time students have to deal with, are the self-organized learning or the (on time) accomplishment of work assignments, which are often perceived as too extensive, and the independent development of teaching content. Furthermore, part-time students reported about the difficultness to reconcile e‑learning and work and/or family.

**Conclusion:**

These perceived challenges are not new but since additional family commitments and uncertainty of daily work routine are added, these challenges are gaining in importance during COVID-19. Therefore, learning environments, which are currently offered to part-time students, must be understood as caring spaces. In addition to new didactic concepts, universities should invest in the promotion of students’ personal and social competencies.

## Einleitung

Im Frühjahr 2020 wurden nicht nur Hochschulen, sondern die gesamte Welt mit dem neuartigen Coronavirus überrascht. Medial verbreitete sich rasch die Meldung, dass österreichische Hochschulen, mit einer einwöchigen Übergangsfrist, ihre Präsenzlehre einstellen und Studierende sowie Lehrende im Sinne des Social Distancing zu Hause bleiben müssen^1^. E‑Learning ist per se nicht neu für Hochschulen, allerdings führte die COVID-19-Pandemie zu einer rapiden Umstellung auf 100 %ige Onlinelehre (Almaiah et al. 2020). Ergebnisse der Onlineumfrage im Auftrag des österreichischen Bundesministeriums für Bildung, Wirtschaft und Forschung zeigen, dass mehr als die Hälfte von 517 befragten Studierenden die Umstellung auf Onlinelehre mit sehr gut (17 %) bzw. gut (44 %) bewerten (Hajek und Kernecker 2020). In der Studie von Schober et al. (2020) berichten knapp 16 % von 2559 Studierenden über Bewältigungsprobleme. In der österreichischen Studierenden-Sozialerhebung (Unger et al. 2020) gaben knapp 65.000 Studierende an, berufsbegleitend zu studieren. Weiters berichteten berufsbegleitend Studierende häufiger, an mindestens einer stressbedingten gesundheitlichen Beschwerde zu leiden, als Studierende im Vollzeitstudium, was mitunter auf die unterschiedliche Gesamtbelastung von Studium und Erwerbstätigkeit zurückzuführen ist. Gerade jetzt muss manchen Studierenden die Vereinbarkeit von 100 %iger Umstellung auf E‑Learning und voller Anwesenheit in einem systemerhaltenden Beruf gelingen. Die Rede ist insbesondere von berufsbegleitend Studierenden, welche etwa im Gesundheitsbereich tätig sind. Die Versorgung von COVID-19-Erkrankten, aber auch der Schutz von Risikogruppen sowie die Vermeidung eines kollabierenden Gesundheitssystems stellen insbesondere Pflegedienstleitungen, aber auch Pflege- und Gesundheitspersonal im stationären und im ambulanten Bereich vor enorme Herausforderungen (Famira-Mühlberger 2020).

Auch ohne COVID-19-Pandemie sehen sich berufsbegleitend Studierende mit Schwierigkeiten bei der Vereinbarkeit von Studium, Erwerbstätigkeit und familiären Verpflichtungen konfrontiert (Brunner und Kada 2011). Einerseits finden oftmals konfliktbehaftete Gespräche mit Vorgesetzten, Kollegium oder Familie statt, und andererseits gilt es, einen positiven Studienerfolg sicherzustellen. Dabei spielen die Anforderungen der Arbeitsstelle und das Ausmaß der Arbeitszeit eine bedeutende Rolle (Buß 2019). Denn erhöht sich das Ausmaß der Arbeitszeit, reduzieren sich die Freizeit und in weiterer Folge auch die Studienzeit, die Teilnahme an Lehrveranstaltungen und die Zeit für studienbezogene Aufgaben (Beerkens et al. 2011). Es kann davon ausgegangen werden, dass sich während der COVID-19-Pandemie die Anforderungen und die Arbeitszeiten im Gesundheitswesen sowie die emotionalen Belastungen erhöhen und damit berufsbegleitend Studierende weniger Zeit für das Selbststudium aufbringen können. Doch welche Herausforderungen die aktuelle Situation für berufsbegleitend Studierenden aus dem Gesundheitsbereich mit sich bringt, ist bis dato kaum unerforscht.

### Forschungsfrage

So ergibt sich die Frage *„Welchen Herausforderungen stehen berufsbegleitend Studierende aus dem Gesundheitsbereich im Hochschulkontext in Zeiten von COVID-19 gegenüber?“* Ziel der Studie ist es, die Herausforderungen zu explorieren, Veränderungsnotwendigkeiten aufzuzeigen und Handlungsempfehlungen für die Hochschule abzuleiten.

## Methodik

### Untersuchungsdesign

Vom 01. April 2020 bis 31. Mai 2020 fand die Onlineumfrage zum Thema „Selbstorganisation und die Herausforderungen von berufsbegleitend Studierenden während der COVID-19 Krise“ statt. Der Link wurde an alle berufsbegleitenden Studierende der Studiengänge Gesundheits- und Pflegemanagement der Fachhochschule Kärnten (FHK) gesendet. Herausforderungen aufgrund der COVID-19-Krise im Hochschulkontext, subjektiv wahrgewonnene Vor- und Nachteile sowie Verbesserungsvorschläge für die Onlinelehre wurden mittels offenem Antwortformat erfragt. Teti et al. (2020) sehen qualitative Zugänge als Mittel der Wahl, um die Bedürfnisse von berufsbegleitend Studierenden aus dem Gesundheitsbereich während Gesundheitskrisen zu erfassen, um in weiterer Folge Präventionsstrategien und Lösungen ableiten zu können. Weiter wurden das Ausmaß der Arbeitstätigkeit, die Anzahl der Kinder, das Geschlecht und die Betreuung von Familienmitgliedern gefährdeter Personengruppe erhoben. Um die Anonymität zu wahren, wurde auf die Altersangabe und Differenzierung nach Bachelor und Master verzichtet.

### Stichprobe

Die hochselektive Stichprobe besteht aus knapp der Hälfte (48 % bzw. 46 Studierende) aller berufsbegleitend Studierenden aus den Studiengängen Gesundheits- und Pflegemanagement (*n* = 96). Unvollständige Fragebögen wurden ausgeschlossen, daher beruhen alle Ergebnisse auf einer Stichprobengröße von *n* = 40. Von den 40 Studierenden waren 34 weiblich. Zum Zeitpunkt der Befragung waren lediglich 4 Personen (10 %) nicht erwerbstätig. Ein Viertel (*n* = 10) arbeitete Vollzeit (100 %), 17,5 % (*n* = 7) in einem Ausmaß von 75 %, 14 Studierende (35 %) gaben an, Teilzeit (50 % Anstellung) zu arbeiten, und 3 Studierende (7,5 %) in einem Ausmaß von 25 %, und zwei Personen gaben an, anderwärtig beschäftigt zu sein. Mehr als ein Viertel (27,5 %) haben Kinder, und mehr als die Hälfte (52,5 %) kümmerten sich zum Zeitpunkt der Befragung um Familienmitglieder, welche zu den gefährdeten Personengruppe während der „Coronakrise“ zählen.

### Datenauswertung

Die Auswertung der qualitativen Daten erfolgte anhand der qualitativen Inhaltsanalyse (Mayring 2015). Dafür wurde ein induktiver Ansatz bei der Bildung von Haupt- und Subkategorien gewählt. Zur Beschreibung und zur Gewährleistung der intersubjektiven Nachvollziehbarkeit wurden für jede Haupt- und Subkategorie eine Definition sowie typische Textpassagen als Ankerbeispiele formuliert. In einem ersten Schritt wurden einzelne Textstellen markiert und erste Hauptkategorien gebildet, welche nach der Hälfte des zu analysierenden Materials auf ihre Sinnhaftigkeit und Trennschärfe geprüft, angepasst bzw. ergänzt wurden.

**Figure 1:**
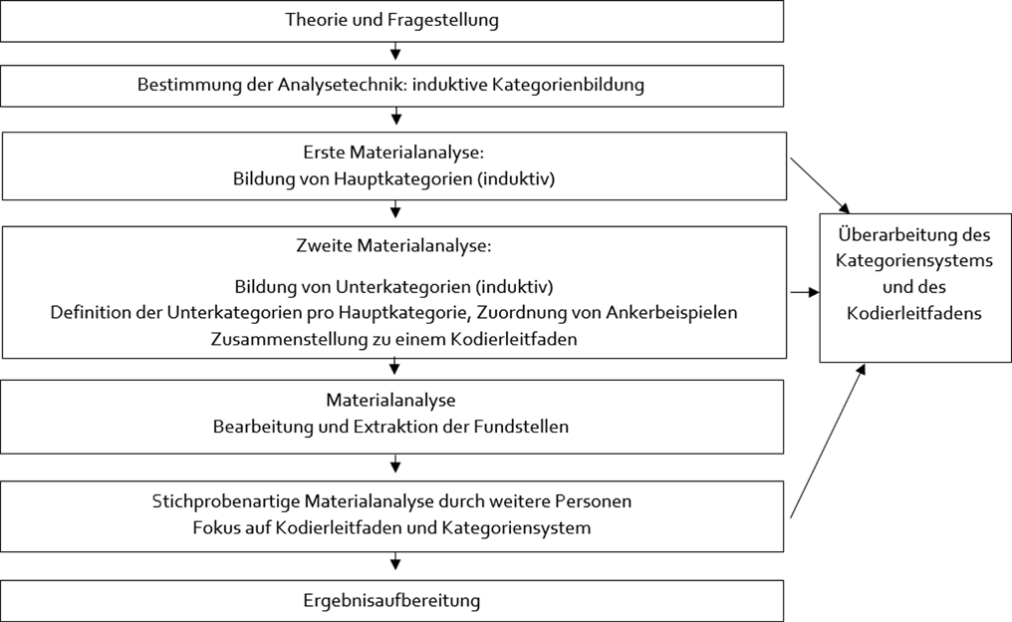

Unterkategorien mit Definitionen und Ankerbeispielen wurden gebildet und der Kodierleitfaden schrittweise generiert. Anschließend wurden weitere Fundstellen extrahiert und den passenden Haupt- bzw. Subkategorien zugeordnet. Für die Vorgehensweise der Auswertung wurde ein Ablaufmodell erstellt (Abb. 1). Durch die regelgeleitete Auswertung und die Erstellung eines Ablaufmodells wird die Nachvollziehbarkeit unterstützt (Brunner 2006). Regelmäßiges Prüfen und Revidieren des Kategoriensystems, Festlegen der Ankerbeispiele und Reflexion der Ergebnisse mit 2 weiteren Personen führten zu reliablen, trennscharfen Kategorien.

## Ergebnisse

Die Studierenden wurden zu ihren aktuellen Herausforderungen befragt, die Aussagen konnten folgenden Hauptkategorien zugeordnet werden: „E-Learning“; „Vereinbarkeit von E‑Learning mit Beruf und/oder Familie“, „Semesterabschluss“, „fehlende Sozialkontakte“ und „Ausmaß der Berufstätigkeit“. Um die Antworten differenzierter betrachten zu können, wurden für einige Kategorien Subkategorien gebildet. Zusätzlich wurde noch die Anzahl der Nennungen (*n*) erfasst, um zu veranschaulichen, wie sich die Antworten auf die Kategorien aufteilen.

Für die Hauptkategorie E‑Learning (*n* = 35) wurden die Subkategorien „selbstorganisiertes Lernen“, „Umstellung auf Onlinelehre“ und „Technik“ gebildet. Die meisten Herausforderungen (*n* = 25) bezogen sich auf das „selbstorganisierte Lernen“. Arbeitsaufträge wurden als zu umfangreich empfunden und die Bewältigbarkeit infrage gestellt. Weiter wurden die Einhaltung von Abgabefristen sowie das selbstständige Erarbeiten von Lehrinhalten genannt (*„Durch das Wegfallen der Präsenzlehre sind Ersatzleistungen zu erbringen, welche neben den regulären Arbeitsaufträgen zu erbringen sind und in keinem Verhältnis zu der Zeiteinheit der weggefallenen Präsenzlehre stehen“, F.45*). Bezugnehmend auf die Umstellung zur Onlinelehre wurden die Umstellung an sich, und das Aufbringen von ausreichendem Engagement als herausfordernd wahrgenommen. In der Subkategorie „Technik“ wurde die Nutzung von unterschiedlichen Kommunikationsportalen als herausfordernd genannt. Die Hauptkategorie „Vereinbarkeit von E‑Learning mit Beruf und/oder Familie“ (*n* = 16) unterteilt sich in die Subkategorien „Beruf und/oder Familie“ und „Homeschooling“. Zu den Herausforderungen zählten, den Anforderungen der Hochschule gerecht zu werden, sowie die Vereinbarkeit von Kinderbetreuung und den vermehrten beruflichen Anforderungen. Weiter wurde von einer vermehrten beruflichen Anwesenheit und einer schlechten Planbarkeit der beruflichen Verpflichtungen berichtet (*„Aktuell viele kurzfristig erforderliche berufliche Tätigkeiten, schlecht planbar“, F.44*). Die Hauptkategorie „Semesterschluss“ (*n* = 13) wurden in die Subkategorien „unklare Prüfungsmodalitäten“ und „fehlende zeitliche Ressourcen für Abschlussarbeiten“ unterteilt. Herausfordernd wurde die Unklarheit über Ablauf und Zeitpunkt von Prüfungen und Zeitmangel, um die Abschlussarbeiten fertigzustellen, empfunden. In der Hauptkategorie „fehlende Sozialkontakte“ (*n* = 4) wurde der fehlende Kontakt zu Mitstudierenden und Lehrenden genannt. Studierende berichteten über vermehrte Überstunden und ein höheres Ausmaß an erlebtem Stress im beruflichen Umfeld – diese Aussagen wurden der Hauptkategorie „Ausmaß der Berufstätigkeit“ (*n* = 3) zugeordnet. Die subjektiv empfundenen Vor- und Nachteile der Online-Lehre werden in Tab. 1 abgebildet.

**Table 1:** 

Vor- und Nachteile der Onlinelehre	
Vorteile	Nachteile
Freier Einsatz von zeitlichen Ressourcen (freie Zeiteinteilung bei der Erledigung von Arbeitsaufträgen und Lehrinhalten, Flexibilität in der Zeiteinteilung, Selbstorganisation bzw. Eigenverantwortung daher beruflich flexibler)	Erhöhter Aufwand und Umfang bei Arbeitsaufträgen
Wegfall der Anreise (Zeit und Geld wird durch den Wegfall gespart)	Diskussionen schwer möglich
Verringerung der Präsenzzeiten	Fehlendes Feedback und Erklärungen von Lehrenden
Fortführung der Lehrveranstaltungen	Wenig Lernerfolg
Förderung der Medienkompetenz	Eingeschränkter Kontakt zu Mitstudierenden und Lehrenden
Verbesserung der Ernährungsgewohnheiten	Verringerte Mimik und Gestik
	Durchführung von Gruppenarbeiten per Mail oder Handy schwierig
	Motivations- und Konzentrationsschwierigkeiten
	Verwendung von unterschiedlichen Plattformen und Medienkanälen
	Fehlende technische Ausstattung
	Unklarer Ablauf von Onlineprüfungen

Hinsichtlich der Verbesserungsvorschläge aus Sicht der befragten Studierenden wäre eine bessere Abstimmung der Arbeitsaufträge, Prüfungen etc. unter den unterschiedlichen Lehrenden wünschenswert. Auch einen angemessenen Zeitraum zur Bearbeitung von Arbeitsaufträgen zu bekommen bzw. Arbeitsaufträge zu reduzieren, ist zu empfehlen. Gruppenarbeiten sollten auf ein Minimum beschränkt werden, da diese aus Sicht der Befragten schwer zu organisieren sind. Studierende wünschen Aufzeichnungen der Lehrveranstaltungen sowie Videos, mehr Erklärungen und Beispiele. Die Nutzung unterschiedlicher Lernplattformen (Moodle, Laufwerke …) soll sich auf eine Plattform beschränken. Hinsichtlich der Prüfungssituation würden sich die Studierenden Ersatzleistungen bzw. eine Reduzierung von Abschlussarbeiten oder das Wegfallen von Zwischenaufgaben wünschen. Mehr Betreuung und Rücksichtnahme vonseiten der Lehrenden sollten Eingang in die Onlinelehre finden.

## Diskussion

Die erwähnten Herausforderungen sind grundsätzlich nicht neu und treffen mitunter auch auf andere (berufsbegleitend) Studierende zu (Kittel und Rollett 2017). Auch Studien abseits von COVID-19 (Diehl et al. 2018; Gerholz und Klingsieck 2013; Hofmann et al. 2017; Stadtfeld et al. 2019) identifizieren Selbst- und Zeitmanagement, soziale Integration bzw. Einsamkeitserleben und Bewältigung von hochschulischen Anforderungen als relevante Themenkomplexe mit Handlungsbedarf. Generell zeigt sich in aktuell durchgeführten COVID-19-Studien in der D‑A-CH-Region, dass Studierende mit einer erhöhten Arbeitslast zu kämpfen haben (Klug und Meister 2020; van de Velde et al. 2021), Unklarheiten in Bezug auf Modulerwartungen sowie eine schlechte Erreichbarkeit von Lehrenden zu Stress führen (Rüegg und Eggli 2020) und auch fehlenden Kontakte zu Mitstudierenden und Dozierenden als belastend eingestuft werden. Elmer et al. (2020) untersuchten in ihrer Longitudinalstudie die Veränderung der psychischen Gesundheit und der sozialen Netzwerke von 212 Bachelor-Studierenden in der Schweiz vor und während der COVID-19-Pandemie. Dabei zeigte sich, dass Interaktionen und Studierendennetzwerke abgenommen haben und Studierende nun eher alleine studieren. Zudem nahmen das Stressempfinden, Ängste und Einsamkeit zu. Sorgen über die Gesundheit, Familie und Freunde und die eigene Zukunft kamen hinzu. Explorative Analysen, die im Rahmen dieser Studie durchgeführt worden sind, legen die Vermutung nahe, dass COVID-19-spezifische Sorgen, die soziale Isolation und damit fehlende Unterstützung mit einem negativen Verlauf der psychischen Gesundheit verbunden sind.

Die aktuelle Lage unter COVID-19 von berufsbegleitend Studierenden, welche in Gesundheitsbereichen tätig sind, ist bis dato kaum erforscht. Die vorliegende Studie konnte aufzeigen, dass die drei größten Herausforderungen, mit denen diese Zielgruppe in Zeiten der COVID-19-Pandemie zu kämpfen haben, folgende sind:

- die Selbstorganisation des Lernens,
- die (termingerechte) Bewältigung der, oft als zu umfangreich empfundenen, Arbeitsaufträge und
- das selbstständige Erarbeiten von Lehrinhalten.

Unter COVID-19 werden diese Themenkomplexe für diese Zielgruppe bedeutsamer denn je, da noch zusätzliche familiäre Verpflichtungen wie Versorgung von Familienmitgliedern aus den Risikogruppen, Homeschooling, aber auch Unsicherheiten im Arbeitsalltag dazukommen. Unsicherheiten ergeben sich durch mögliche Dienständerungen, wenn z.B. Pflegepersonal von „COVID-freien“ Stationen auf COVID-19-Stationen oder „intermediate care units“ (ICU) (Brand 2020) eingesetzt wird. Des Weiteren führen der Einsatz von festen Pflegeteams (WKO 2020), die Bereitstellung von Personen als Ersatzpersonal in Alten- und Pflegeheimen (Golla 2020), und auch Überstunden zu einer schlechteren Planbarkeit des Arbeitsalltags beziehungsweise zu vermehrten arbeitsbezogenen Belastungen. Hinzu kommen noch eine reduzierte soziale Unterstützung infolge von langen Arbeitszeiten und auch Stigmatisierung in Zeiten von COVID-19. Auch eine Verringerung der Selbstfürsorge aufgrund von Zeit- und Energiemangel spielt eine Rolle (Petzold et al. 2020). Weiters führen Petzold et al. (2020) aus, dass es aufgrund von Trennungen innerhalb des Teams, in dem üblicherweise zusammengearbeitet wird, oder eines schlechten Gewissens, wenn Kolleg*innen mit zusätzlicher Arbeit konfrontiert werden, zu einem Gefühl von sozialer Isolation kommt. Kühlmeyer et al. (2020) berichten über die Konfrontation mit belastenden moralischen Herausforderungen (z. B. aufgrund der Versorgung von COVID-19-Patient*innen erhöht sich die Gefahr, sich selbst und in weiterer Folge sein persönliches Umfeld anzustecken, was zu einer sozialer Isolation, Stigmatisierung aus dem eigenen Umfeld und zu einer Selbstdistanzierung im Kontakt zu erkrankten Patient*innen führen kann) von Medizinstudierenden und ärztlichen Berufseinsteigenden. Fehlen den Studierenden allerdings Bewältigungsstrategien im Umgang mit solch moralisch herausfordernden Situationen, können sie sehr schnell in eine vulnerable Situation fallen. Aus Studien vorangegangener Gesundheitskrisen wie A/H1N1-Influenza (Goulia et al. 2010; Matsuishi et al. 2012) oder SARS (Goulia et al. 2010; Matsuishi et al. 2012; Nickell et al. 2004) ist bekannt, dass Gesundheitskrisen mit einem erhöhten Stresslevel, Belastungen und einer Verschlechterung der psychischen Gesundheit von Gesundheits- und Pflegepersonal einhergehen. In der Studie von Li et al. (2020) zeigten 26,63 % von 1442 Studierenden aus Gesundheitsstudiengängen klinische Symptome von Disstress, bzw. 11,10 % litten unter einer akuten Stressreaktion während der COVID-19-Pandemie. Umso wichtiger ist es nun, berufsbegleitend Studierende aus dem Gesundheits- und Pflegebereich adäquat zu unterstützen (Brand 2020).

Gerade jetzt erscheint für Hochschulen der richtige Zeitpunkt, im Sinne eines studentischen Gesundheitsmanagements (Techniker Krankenkasse 2019), Interventionen auf Studierenden‑, Lehrenden- und Strukturebene zu setzen. Davon können nicht nur berufsbegleitend Studierende, welche in Gesundheitsberufen tätig sind profitieren, sondern es bietet sich dadurch für alle Studierenden die Möglichkeit, bei der Bewältigung hochschulischer An- und Herausforderungen zu unterstützen und ihre Gesundheit zu fördern. Auf der Ebene der Studierenden ist insbesondere die Förderung von Selbst- und Sozialkompetenzen zu adressieren, wie auch die Ergebnisse der vorliegenden Studie unterstützen. So wurden etwa an der FHK im Rahmen des Projekts Kompetent und kohärent im Studium-Toolbox (KukiS-Toolbox) wissenschaftlich fundierte Lehr- und Lernmaterialien zur Förderung von Selbst- und Sozialkompetenzen bei Studierenden entwickelt. Eine wichtige Rolle im Zusammenhang mit Selbstkompetenzen spielt das Kohärenzgefühl – das Vertrauen in die eigene Fähigkeit, eine stressvolle und unerwartete Situation bewältigen zu können (Antonovsky 1979). Ein gut ausgeprägtes Kohärenzgefühl lässt Situationen als verstehbar, sinnhaft und bewältigbar erscheinen und trägt zu einer effizienteren Erholung von stressvollen Situationen bei (Lundberg und Toivanen 2019). Die erfolgreiche Bewältigung von Belastungen und Stress geht mit einer positiven Kompetenzerfahrung einher und wirkt gesundheitsfördernd (Franke und Witte 2009). Eine klare einheitliche Kommunikation über aktuelle COVID-19-Maßnahmen an der Hochschule, eine klare und strukturierte Einteilung der Prüfungstermine und des Prüfungsablaufes, gepaart mit einer frühzeitigen Kommunikation darüber, fördern die Verstehbarkeit und Bewältigbarkeit der Studierenden. Derartige strukturelle Maßnahmen schaffen transparente Vorgaben als Ausgangspunkt für die individuelle Erstellung von Zielen und Handlungsplänen und stärken das Kohärenzgefühl. So kann auch akademische Prokrastination angesprochen und unterbunden werden. Allgemeine Tipps dazu finden sich beispielsweise in der Broschüre *Willi will BALD* (https://blog.fh-kaernten.at/kukis/files/2019/12/Folder-Prokrastination.pdf). Speziell für die COVID-19-Situation wurde ein Factsheet zum Thema „Lernen in Zeiten von Corona“ (https://blog.fh-kaernten.at/kukis/files/2020/03/Lernen-in-Zeiten-von-Corona_final.pdf) erstellt.

Wesentlich in der Bewältigung von An- und Herausforderungen ist soziale Unterstützung. Beziehungen gestalten und aufrechterhalten zu können sind bedeutsame Voraussetzungen, um in weiterer Folge soziale Unterstützung aktivieren zu können (Dassler 2009). Damit ist eine Fokussierung auf Sozialkompetenzen unausweichlich. Beispielsweise konnte während der SARS-Epidemie aufgezeigt werden, dass insbesondere „peer support“ bei in Gesundheitsberufen Tätigen das Risiko von stressbezogenen Symptomen reduziert (Shapiro und Galowitz 2016) und auch bei Studierenden mit psychischen Problemen als Interventionsansatz hilfreich ist (Huang et al. 2018). Laut McIntyre et al. (2018) sind hochschulische freundschaftswertige Beziehungen einer der effektivsten Protektoren, um Stress vorzubeugen. Im Rahmen der KukiS-Toolbox wurde ein Factsheet zum Thema „Tipps gegen Einsamkeit in Zeiten von Social Distancing“ erstellt (https://blog.fh-kaernten.at/kukis/beispiele-aus-der-toolbox-2/tipps-gegen-einsamkeit-in-zeiten-von-social-distancing/). Auch Lehrende sind hier gefordert, neue kreative (Online‑)Räume zu schaffen, um soziale Unterstützung zu bieten und das soziale Miteinander zu fördern. Es geht darum, ein Gemeinschaftsgefühl zu erzeugen, Verbundenheit zueinander und zur Hochschule als Institution als bedeutsame Grundlage von studentischem Commitment in den Fokus zu nehmen. So müssen Lernumwelten, wie sie Hochschulen berufsbegleitend Studierenden derzeit online bieten sollen, als „caring spaces“ verstanden werden (Samuel 2017): Respekt, Anerkennung der Arbeits- und Lebensrealitäten und deren Auswirkungen auf das Lernen sollten Raum zur gemeinsamen Reflexion erhalten. Lehrende sollen bedeutsame Geschehnisse mit den Studierenden thematisieren und Studierende, mit all ihren Sorgen und Ängsten, aber auch mit ihren Kompetenzen, in einem holistischen Bild sehen (Limarutti und Mir 2020). Grundsätzlich bietet Onlinelehre eine gute Möglichkeit, die Vereinbarkeit zwischen Studium und dem Rest zu schaffen. Allerdings gelingt dies nur mit einem adäquaten didaktischen Konzept, welches Inhalte, Ziele und Rahmenbedingungen des Lehr- und Lernsettings berücksichtigt und sich an den Bedürfnissen von Lehrenden und Lernenden orientiert (Hawlitschek und Fredrich 2018). Wie kann dies nun exemplarisch gelingen? Lehrveranstaltungskonzepte, die dem „online inverted classroom model“ (oICM, Tolks et al. 2020) folgen, sind hier ein guter Ansatz. Dabei wechseln synchrone Onlinetermine im „Digi-Audimax“ mit asynchronem Onlineselbststudium ab. Im Zuge des asynchronen Onlineselbststudiums können sich Studierende zeit- und ortsunabhängig aktiv Wissen erarbeiten, was im Sinne der Vereinbarkeit der Lebenswelten einen großen Vorteil bietet. Im Rahmen synchroner Onlinetermine, unter Einsatz von „Audience-response“-Systemen kann der Wissenserwerb überprüft werden, und die Studierenden erhalten Feedback zu ihren Lernfortschritten. Dadurch können Probleme im Selbstmanagement der Studierenden frühzeitig erkannt und aktiv adressiert werden. Durch das gemeinsame Vertiefen des Erlernten im „Digi-Audimax“ und das kontinuierliche Einholen von studentischem Feedback zur Lehrveranstaltung wird ein Raum für inhaltliches und soziales Miteinander gestaltet. Als Lehrende präsent und ansprechbar zu sein, ist in Zeiten digitaler Lehre wichtiger denn je. Der Einsatz von Foren oder auch die Etablierung von Onlinesprechstunden stellen hier wichtige Werkzeuge dar. Neben der Präsenz der Lehrenden gilt es auch, die studentische Partizipation bei der Lehrveranstaltungsdurchführung zu stärken. Teilhabe kann Verbundenheit fördern und so wesentlich zum Lernerfolg der Studierenden beitragen. So können etwa die Generierung möglicher Prüfungsfragen von den Studierenden selbst, das Bearbeiten von Aufgabenstellungen in „Breakout“-Gruppen (synchron online) oder auch der kontinuierliche Einsatz von Audience-response-Systemen die studentische Partizipation unterstützen. Damit wird Onlinelehre in Zeiten der Coronapandemie zu einer großen didaktischen Herausforderung, die neue, lustvolle Wege des gemeinsamen Lehrens und Lernens eröffnet. Ein positives „mindset“ gegenüber dem veränderten Miteinander zu schaffen, gehört dabei ebenso zu den Aufgaben von Lehrenden. Um die Vorhersagbarkeit und Bewältigbarkeit von Lehrveranstaltungen zu erhöhen, bedarf es von Anbeginn an einer klaren Kommunikation über das Programm – ein „onboarding“ mit Zieldefinition, strukturierter Vorstellung der synchronen und asynchronen Anteile sowie transparenter Darlegung der Prüfungsbedingungen ist dabei unerlässlich. Auf Lehrendenebene sind es somit 4 Grundprinzipien, die gerade berufsbegleitend Studierende in Zeiten der Coronapandemie unterstützen können. Diese kann man plakativ mit den „4 Ps der Onlinelehre“ zusammenfassen: positives Mindset, Programm, Partizipation und Präsenz.

Neben der Frage, was Studierende und Lehrende tun können, um die veränderten Lehr- und Lernbedingungen positiv nutzen zu können, gilt es auch, strukturelle Veränderungsnotwendigkeiten in den Blick zu nehmen. Was können etwa Hochschulen als Institution tun?

Um gesundheitsförderliche Interventionen, die zur Bewältigung der genannten Herausforderungen und zur Förderung der Studierfähigkeit und individuellen Gesundheit beitragen, verankern zu können, wird etwa an der FHK im Rahmen des Projekts SHAB@CUAS^2^ (Student Health Advisory Board at Carinthia University of Applied Sciences) ein Gesundheitsbeirat, bestehend aus einer heterogenen Gruppe an Studierenden, initiiert. Dieser innovative partizipative Ansatz bietet die Möglichkeit, studentische Entscheidungsfindung und Selbstorganisation in Bezug auf Studierendengesundheit zu stärken. Dieser Gesundheitsbeirat treibt gesundheitsfördernde und COVID-19-relevante Themen an der FHK voran, indem Interventionen geplant, pilotiert und evaluiert werden und somit handlungsleitende Elemente für eine nachhaltige Implementierung entwickelt werden können.

## Schlussfolgerung

In Gesundheitsberufen tätigen, berufsbegleitend Studierenden kommt im Rahmen der aktuellen Coronapandemie eine besondere Rolle zu. Einerseits helfen sie bei der Bewältigung dieser Krise, und anderseits sind sie damit beauftragt, studienrelevante Anforderungen und Aufgaben abzuarbeiten und eine Balance zwischen Beruf und Privaten zu finden. Diese empfundenen Herausforderungen sind grundsätzlich nicht neu und treffen mitunter auch auf andere berufsbegleitend Studierende zu, allerdings rücken sie aufgrund der Pandemie vermehrt in den Vordergrund. Nun erscheint der richtige Zeitpunkt, sich dieser Herausforderungen bewusst zu werden und zu handeln und Interventionen auf Studierenden‑, Lehrenden- und Strukturebene zu setzen. Davon können nicht nur berufsbegleitend Studierende, welche in Gesundheitsberufen tätig sind, profitieren, sondern es unterstützt alle Studierende bei der Bewältigung hochschulischer An- und Herausforderungen und fördert ihre Gesundheit. Im Rahmen des Beitrags wurde versucht, erste mögliche Lösungsansätze auf allen drei Ebenen für die aktuelle Situation zur Verfügung zu stellen. Studierenden sollen im Bereich ihrer Selbst- und Sozialkompetenzen gefördert werden. Dabei sind insbesondere das Selbstmanagement, das Kohärenzgefühl und die soziale Unterstützung zu fokusieren. Auch Lehrende sollen Studierenden neue (Online‑)Räume schaffen, um das soziale Miteinander zu fördern. Zudem können gute Onlinelehrkonzepte (z. B. oICM) die Vereinbarkeit von Beruf und Studium erleichtern. Letztlich geht es auch um eine strukturelle Einbettung von Interventionen in das System Hochschule. Denn die Förderung eines breiteren akademischen Interesses und das dauerhafte Engagement in Gesundheitsförderung leisten einen Beitrag zur Zielerreichung im Sinne des studentischen Gesundheitsmanagements (Techniker Krankenkasse 2019) und des Ansatzes der „healthy universities“ (Holt et al. 2015).

## Limitationen

Herausforderungen von berufsbegleitend Studierenden aus dem Gesundheitsbereich sind bis dato ein wenig beforschtes Feld. Obwohl zur Exploration ein qualitativer Zugang als Mittel der Wahl gesehen wird (Niederberger und Dreiack 2020), wird mit der vorliegenden Studie lediglich das subjektive Empfinden einer hochselektiven kleinen Gruppe von in Gesundheitsberufen tätigen berufsbegleitend Studierenden erfasst. Daher sind die Ergebnisse mit Vorsicht zu verstehen, und Generalisierungen sowie eine Übertragung der Ergebnisse auf andere Zielgruppen können, wie meist in der qualitativen Forschung, nicht vorgenommen werden. Zukünftige Untersuchungen sollen Belastungen und posttraumatische Symptomatik bei berufsbegleitend Studierenden mittels standardisierter Erhebungsinstrumente erfassen, um mögliche Folgen der Pandemie sowie Handlungs- und Präventionsansätze für größere Kollektive ableiten zu können.

## Footnotes

1 Regierung verhängt wegen Corona starke Einschränkungen – Coronavirus – derStandard.at › Panorama.

2 Fördergeber*in: Ludwig Boltzmann Gesellschaft (LBG GmbH), Nationalstiftung für Forschung, Technologie und Entwicklung.
